# Ensuring Homogeneity in Powder Mixtures for Pharmaceuticals and Dietary Supplements: Evaluation of a 3-Axis Mixing Equipment

**DOI:** 10.3390/pharmaceutics13040563

**Published:** 2021-04-16

**Authors:** Bruna Marianni, Hudson Polonini, Marcone A.L. Oliveira

**Affiliations:** 1 Fagron BV, Lichtenauerlaan 182, 3062 ME Rotterdam, The Netherlands; hudson.polonini@fagron.com; 2 Department of Chemistry, Federal University of Juiz de Fora, 36036-900 Juiz de Fora-MG, Brazil; marcone.oliveira@ufjf.edu.br

**Keywords:** solid–solid mixing, static mixers, mixing devices, powder mixing, pharmaceutical operations, compounding techniques

## Abstract

The mixing process plays a pivotal role in the quality of pharmaceuticals and food/dietary supplements, as it can impact the homogeneity of the substances in their dosage form and affect characteristics such as dissolution and stability. Thus, the choice of the right mixing device is paramount for compounding pharmacies. In this paper, we evaluated the mixing efficacy of a new 3-axis mixer device and determined its optimal working conditions. Three different formulations were compounded with the device and a total of 540 individual assays were performed by HPLC or ICP-MS to validate its use, in addition to a direct comparison among it and two alternative mixing methods. The 3-axis mixer device was able to provide homogeneous mixtures and finished capsules with adequate content uniformity with a broad range of conditions of use (mixing times from 2 to 8 min, and speed of rotation from 10 to 100 rpm). In addition, the device was superior to classical mixing methods (such as the use of manually shaken plastic bags) and at least equivalent to well-established ones (Y-shaped mixer). Finally, we proposed a cleaning procedure that was also adequate to prevent cross-contamination among products compounded with the same device.

## 1. Introduction

The mixing process plays a fundamental role in the quality of pharmaceuticals and food/dietary supplements, as tit can impact the homogeneity of the substances (which can contain up to 20 different ingredients) in their dosage form and also affect characteristics such as dissolution and stability (in the function of the distribution of functional excipients, such as lubricants and disintegrating agents) [1,2].

One can say that the main, directly objective goal of the different mixing processes available in the market is to provide homogeneity to a given product, to ideally consume a minimum quantity of energy and time, and to have good productivity with the lowest possible cost [3,4]. In this sense, a mixture can be considered homogeneous when any fraction of it contains all components in the same proportion as the total preparation [5].

**Figure 1:**
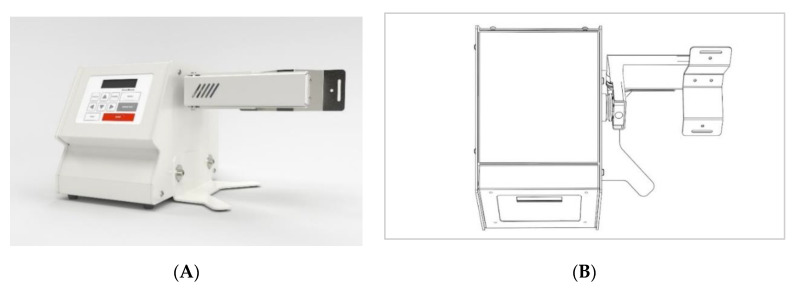
(**A**) 3-axis mixer front side picture (**B**) 3-axis mixer top side scheme (right).

Pharmaceutical industries need to provide evidence of the validation of the production process, and this includes objective parameters that attest to the homogeneity of the mixtures and the consequent uniformity of the final dosage forms. On the other hand, the vast range of possible products produced by compounding pharmacies can pose a challenge for the pharmacist to choose the most adequate method for the pharmacy. Traditionally, the mixture in the compounding pharmacies context for solid dosage forms (notably capsules) is made with mortar and pestle. However, alternative methods are also available, such as the use of small-volume V-shaped or Y-shaped mixers, or even the rudimentary use of manually agitated plastic bags [5,6]. Recently, a new device for powder mixing entered the market—a 3-axis mixer device (FagronLab InvoMatic, Scheßlitz, Germany) (Figure 1).

The choice of the right mixer device is paramount for the compounding pharmacies, as the quality of the mixture highly depends on it. This choice needs to be made through scientifically-based tests that show that any small amount of the sample from a bulk powder represents the physical and chemical characteristics of the entire bulk, and therefore, all dosage forms present the same amount of the active pharmaceutical ingredient (API) [2,7].

As the 3-axis mixer is a new mixing method that has not yet been fully evaluated and, as it claims to be time-saving for the compounding pharmacies, this study was designed to evaluate whether this device can be of added value for the mixing process on a small scale and provide homogeneous mixtures with small, required mixing times.

## 2. Materials and Methods

### 2.1. Materials and Equipment

The 3-axis mixer device evaluated was the FagronLab InvoMatic (Scheßlitz, Germany). All active pharmaceutical ingredients (finasteride, vitamin B12, folic acid, copper chelate, magnesium citrate, and zinc chelate) and hard-shell capsules were obtained from Fagron Brasil (São Paulo, Brazil), and the standardized excipient (Celulomax HG) was obtained from Excipienta (São Paulo, Brazil). High-performance liquid chromatographic (HPLC)-grade reagents (Panreac, Barcelona, Spain) were used; 1% nitric acid Suprapur^®^ was purchased from Merck (Darmstadt, Germany); ultra-pure water was obtained with an AquaMax-Ultra 370 Series (Young Lin, Anyang, Korea) (18.2 MΩcm resistivity at 25 °C) and was used throughout the experiments. The reference standards used were all working standards obtained from primary USP (Rockville, MD, USA) reference materials. Certified reference materials (CRM) for ICP-MS analyses were from NSI Lab Solutions (Raleigh, NC, USA). All volumetric glassware and the analytical balance used were calibrated. HPLC mobile phases were filtered through a 0.45 μm filter membrane (RC-45/15 MS; Chromafil, Düren, Germany) and degassed using an ultrasonic apparatus (Model 1600A; Unique, Indaiatuba, Brazil) for 30 min immediately before use.

HPLC analyses for finasteride, folic acid, and vitamin B12 were performed on a qualified and calibrated chromatography system (Young Lin, Anyang, Korea) composed of a quaternary gradient pump (YL 9110), a photodiode array (PDA) detector (YL 9160), a 96-vial programmable autosampler (YL 9150), a column oven compartment (YL 9130), a variable sample loop up to 200 mL, and a software controller (Clarity). Chromatographic conditions for finasteride quantification were as follows: column—C18 4.6 × 100 mm, at 45 °C; mobile phase—phosphoric acid 2.5mM and acetonitrile (1:1); flux—1.5 mL/min; volume of injection—20 µL; and UV detection at 240 nm. For vitamin B12, the conditions were as follows: column—C18 4.6 × 150 mm, at 25 °C; mobile phase—methanol and water (35:65); flux—0.5 mL/min; volume of injection—100 µL; and UV detection at 361 nm. For folic acid, the conditions were as follows: column—C8 4.6 × 250 mm, at 25 °C; mobile phase—methanol and phosphate buffer (12:88); flux—0.9 mL/min; volume of injection—20 µL; and UV detection at 280 nm.

Copper (Cu), magnesium (Mg), and zinc (Zn) quantification was performed using mass spectrometry with inductively coupled plasma (ICP-MS) (7700x, Agilent, Tokyo, Japan), using argon flux = 15 L min^−1^; plasma frequency = 26.99 MHz; no gas mode (no collision cell used); sample uptake = 40 s at 0.3 rps; rinse between samples = 30 s with water at 0.5 rps, followed by 30 s with 1% nitric acid at 0.5 rps. Tuning solution, blanks, and calibration checks were performed to guarantee accuracy.

### 2.2. Methods

#### 2.2.1. Evaluation of the 3-Axis Mixer Device and Definition of Working Optimal Conditions

The efficacy of the 3-axis mixer device was evaluated through its capacity to produce homogeneous powder mixtures for hard-shell capsules filling. In this sense, the mixtures obtained were encapsulated, and then the outcome evaluated was the content uniformity (CU), using the official methods described in the United States Pharmacopeia [8] and the European Pharmacopoeia [9]; for that, 10 units of each capsule formulation were individually assayed. According to both references, the individual values are used to calculate the acceptance value (AV) using the formula $AV=\left|M-\bar{x}\right|+ks$, in which $\bar{x}$ is the mean of individual contents, expressed as a percentage of the label claim; *M* = $\bar{x}$ for the cases where 98.5% ≤ $\bar{x}$ ≤ T, *M* = 98.5 if $\bar{x}$ < 98.5, and *M* = T, if $\bar{x}$ > T, where T is the target content per dosage unit, expressed as a percentage of the label claim; *k* is the acceptability constant (=2.4); and *s* is the sample standard deviation. To ensure the consistency of the dosage units, the AV should be ≤15.0.

Three different capsule formulations were used for this evaluation, chosen to be representative of the different types of powders: F1 as a model for capsules with low-dosage APIs, i.e., small volumes to mix (finasteride—1 mg; capsule #4); F2 for capsules with more than one API and with different doses and granulometry between them (vitamin B12—5 mg + folic acid—50 mg; capsule #3); and F3 for multicomponent capsules, commonly found for dietary supplements or orthomolecular prescriptions (copper chelate—1 mg + magnesium citrate—150 mg + zinc chelate—25 mg; capsule #00). The total volume of the powders was set in order not to surpass 70% of the nominal capacity of the mixing containers for each device.

In addition to the determination of the AV for each API in each formulation, a design of experiment (DOE) was performed to understand the homogenization process of the device, as well as to attempt to define the optimal working conditions. The selected variables were the mixing time (min) and speed of rotations (rpm), through the 3^2^ factorial design, that is, two factors (mixing time and speed) and three levels (low: −1, central point: 0, and high: +1), which was randomly conducted in a total of 90 experiments, since each level had 10 replicates (Table 1). In all experiments, the use of four porcelain spheres inside the jars was set as a fixed condition, as well as the weighing enough powder to produce 30 capsules in each experiment (from which 10 were randomly chosen for the CU determination). The results from the experiments were used to obtain a response surface, used to calculate the theoretical optimal conditions.

#### 2.2.2. Comparison with Other Devices

After setting the working conditions for the 3-axis mixer, a comparison study was conducted. For that, the capsules were compounded again using the optimal condition previously defined for the device, and two alternative methods were used to verify which one provided the best homogeneity (in terms of the AV) for the formulations. The selected methods were a comparable size bench-top Y-mixer (Brazil), a traditional mixing method used in compounding pharmacies and pharmaceutical industries [6], and the plastic bag method (25cm × 14.5cm), a rustic but still used method for small-volume formulations, in which the powders are placed into the bag and manually shaken. Mixing times for the three methods were the same to allow for comparisons, and the speed of rotation was also kept the same for the 3-axis mixer and the Y-mixer.

#### 2.2.3. Cross-Contamination Evaluation

Additionally, the cleaning procedure of the 3-axis mixer jars and porcelain spheres was evaluated. For this, the jar was washed with water and neutral detergent, then rinsed with 70% ethanol; the porcelain spheres were brushed with water and neutral detergent, and then rinsed with 70% ethanol. After this process, a swab soaked in 92% ethanol was spread on the jar or spheres surfaces, then placed in a test tube and diluted with 5 mL of 92% ethanol for the extraction of the APIs. This solution was then analyzed by HPLC or ICP-MS.

## 3. Results and Discussion

### 3.1. Evaluation of the 3-Axis Mixer Device and Definition of Working Optimal Conditions

Results for the 90 assays performed for each API are described in Table 2 (a total of 540 assays for this first step), as well as their AVs. Defining and ensuring homogeneity in powder mixtures is crucial, as in the vast majority of the solid oral pharmaceuticals and dietary supplements, the APIs are only a small proportion of the entire mass of the dosage form, the rest being composed of excipients [10]. To obtain the continuous, beneficial effect of the products, every dosage form needs to contain the same amount of ingredients, and then a mixture can be defined as homogenous if every sample of it has the same quantitative composition as the others [4].

As the majority of the United States Pharmacopeia [8] and European Pharmacopoeia [9] monographs consider the acceptance range for finished products to be not less than 90% and not more than 110% of the labeled amount of the API, this was also considered to be indicative of the efficiency of the mixing process. In this scenario, in a general manner, all conditions tested (mixing time: from 2 to 8 min; speed range: from 10 to 100 rpm) were able to produce homogeneous powder mixtures, as can be evidenced by the CU of the capsules produced with such mixtures. The exceptions were experiments 6 and 7, and only for folic acid. Interestingly, this did not follow any noticeable trend, as experiment 6 used the highest level for mixing time and the average level for speed, while experiment 7 used the lowest level for mixing time and the highest level for speed. It is also worth remarking that the finished products presented adequate content uniformity even with small mixing times, such as 2 min. With this remark in mind, the experiments still provide a basis for the broad range of utilization of the device and a good cost-benefit, as capsules with API content from 90–110% of the labeled amount could be obtained even at a small speed and short mixing time conditions, making it ideal equipment for the routine of, for example, a compounding pharmacy, which needs to produce a broad range of products every day.

Although there was a general positive result for all the tested ranges of conditions, it is possible to observe that some formulations were more homogeneous than others, for example, comparing the finasteride results and the multimineral results. This can be explained because the powders can have different particle size and size distribution, density, surface morphology, and particle shape, which are some of the factors that influence powder flow [11]. In addition, parameters such as moisture and temperature can also play a role in flowability [7,12]. In this sense, the F2 formulation contained very different APIs: one amorphous (folic acid) and another crystalline (vitamin B12); because of this, the porcelain spheres were added to help reduce the particle size, so that they could mix better as a function of their size.

The spheres are important because the shape variations in powders are relevant, as they can range from very irregular particles to almost spheres or well-defined crystals [13]. In addition, a good number of powders present cohesive properties and tend to agglomerate because of the exposure to a moist atmosphere [14]. Then, the impact of the porcelain spheres against the walls of the jars, with the powder particles in-between, can help in breaking down bigger particles or aggregates, which is desirable once materials with similar particle size and shape tend to form more uniform mixtures [4].

In sequence, the results presented in Table 2 were modeled and, after multiple regression analyses through the least-squares method were performed, the following models were obtained:

y’=103.00 (±0.54) − 0.15 (±0.29) X_1_ − 0.16 (±0.29) X_2_ + 0.91 (±0.51) X_1_^2^ − 1.06 (±0.51) X_2_^2^ − 0.77 (±0.36) X_1_X_2_ (1)

y’=106.78 (±0.56) − 1.51 (±0.30) X_1_ − 3.22 (±0.30) X_2_ + 0.85 (±0.52) X_1_^2^ − 3.40 (±0.53) X_2_^2^ − 1.76 (±0.37) X_1_X_2_ (2)

y’=98.00 (±0.76) + 1.04 (±0.41) X_1_ − 1.74 (±0.41) X_2_ − 2.44 (±0.71) X_1_^2^ + 0.67 (±0.72) X_2_^2^ + 2.54 (±0.50) X_1_X_2_ (3)

y’=104.00 (±0.96) + 0.46 (±0.52) X_1_ − 2.21 (±0.52) X_2_ + 0.95 (±0.90) X_1_^2^ − 1.13 (±0.92) X_2_^2^ + 1.24 (±0.64) X_1_X_2_ (4)

y’=107.85 (±0.60) + 0.22 (±0.33) X_1_ − 1.76 (±0.33) X_2_ + 2.35 (±0.57) X_1_^2^ − 1.37 (±0.57) X_2_^2^ + 0.01 (±0.40) X_1_X_2_ (5)

y’=101.06 (±0.90) + 0.53 (±0.49) X_1_ − 1.48 (±0.49) X_2_ + 4.28 (±0.85) X_1_^2^ − 1.46 (±0.86) X_2_^2^ + 0.34 (±0.60) X_1_X_2_ (6)

where Equation (1) is finasteride, Equation (2) is vitamin B12, Equation (3) is folic acid, Equation (4) is copper chelate, Equation (5) is magnesium citrate, Equation (6) is zinc chelate, X_1_ represents the mixing time, and X_2_ is the speed of rotation.

**Figure 2:**
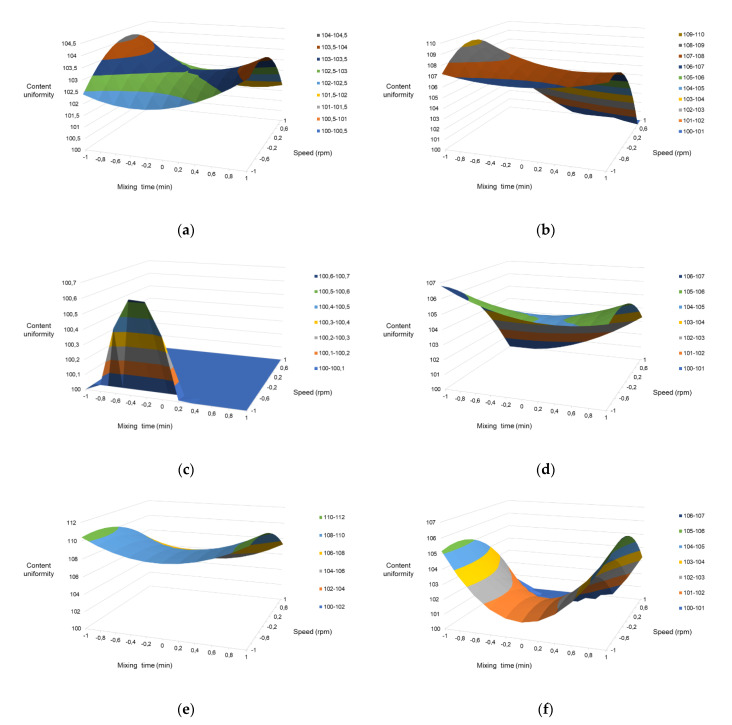
Surface responses. F1: (**a**) finasteride capsules; F2: (**b**) vitamin B12 and (**c**) folic acid capsules; F3: (**d**) copper chelate, (**e**) magnesium citrate, and (**f**) zinc chelate capsules.

This model showed no evidence of lack of fit within the 95% confidence interval for the samples tested, once the lack of first tests returned the F_calculated_ value as lower than the F_critical_ value. Thus, the response surfaces (Figure 2) were built. Observation of the response surfaces allows for the interpretation that the responses tended to increase or decrease under the central points, following opposite outcomes in the extremities (lower or higher levels). Nevertheless, they confirmed that the full range tested could potentially provide results with acceptable AVs and capsule content (90–100%).

### 3.2. Comparison with Other Devices

**Figure 3:**
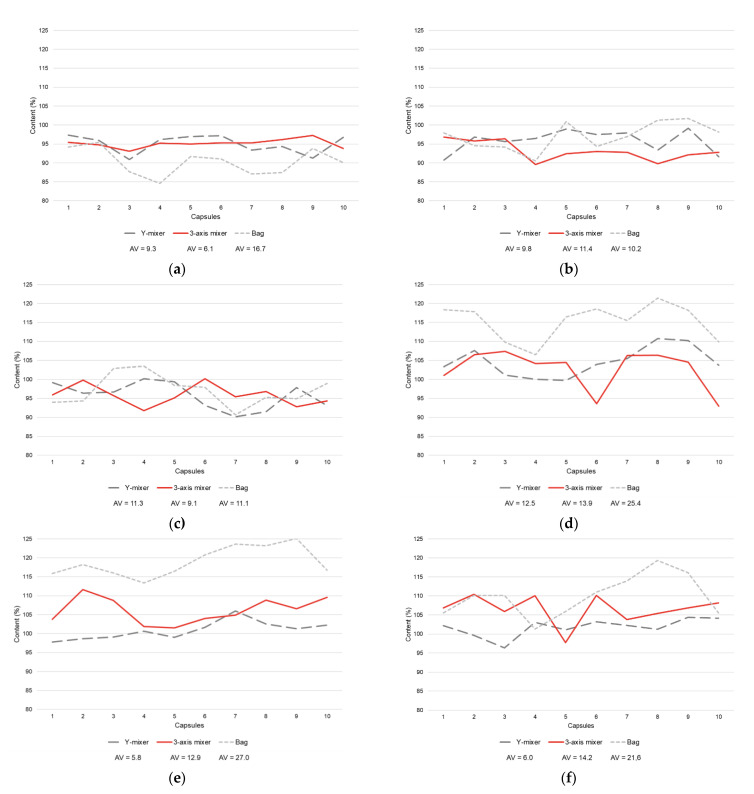
Comparison of the use of the 3-axis mixer with other common methods used for homogenization in compounding pharmacies. F1: (**a**) finasteride capsules; F2: (**b**) vitamin B12 and (**c**) folic acid capsules; F3: (**d**) copper chelate, (**e**) magnesium citrate, and (**f**) zinc chelate capsules.

On the basis of the surface responses and the individual values obtained from each experiment, specific conditions were defined to be fixed and used to provide a comparison of the homogenization efficiency among the 3-axis mixer and the alternative methods (Y-mixer and plastic bag): for F1, X_1_ was at −1 and X_2_ was at 0 (2 min, 50 rpm); for F2, X_1_ was at 0 and X_2_ was at −1 (5 min, 10 rpm); and for F3, X_1_ was at +1 and X_2_ was at 0 (8 min, 50 rpm) (Figure 3). Those different conditions were used to validate that any condition on the tested range could lead to acceptable AVs. Alternative methods were tested using the same mixing time, and Y-mixer was operated using the same speed of rotation.

The results showed that the use of the 3-axis mixer was superior, or at least comparable, to the alternative methods (Figure 3) through the evaluation of the content uniformity among the capsules. Additionally, it was noticeable that the 3-axis mixer and the Y-mixer presented more similar results than the ones obtained from the plastic bag, showing that automation increases the reliability of the mixing process. This can be understood by the Van der Waals forces, which affect the flowability of powders due to adhesive forces between individual particles [12,15]. The surfaces of different materials can share their electric charge, from one surface to the other, during the mixing process, but when the process stops some of the charges may not flow back to the original surface. This results in electrostatically charged particles, leading to a lack of homogeneity in solid formulations. In this context, the friction between the powder particles and the plastic surface can aggravate this process [5,16].

### 3.3. Cross-Contamination Evaluation

The evaluation of the jars and spheres after the compounding and cleaning procedures proved that the cleaning method used was able to reduce the API amounts on their surface to non-detected levels by HPLC (limits of detection = 2.50 µg mL^−1^ for finasteride, 0.055 µg mL^−1^ for vitamin B12, 6.59 µg mL^−1^ for folic acid, 0.01 ng L^−1^ for Cu, 4.0 ng L^−1^ for Mg, and 0.4 ng L^−1^ for Zn). This confirmation provides additional safety validation for using the 3-axis mixer device, as cleaning the critical parts that contact the API is sufficient to prevent cross-contamination among different formulations produced.

## 4. Conclusions

On the basis of the outcomes of this study, we can conclude that the 3-axis mixer device was able to provide homogeneous mixtures and finished capsules with adequate content uniformity with a broad range of conditions of use (mixing times from 2 to 8 min, and speed of rotation from 10 to 100 rpm) regarding the tested formulations. In addition, the device was superior to classical mixing methods (such as the use of manually shaken plastic bags) and equivalent to well-established ones (Y-shaped mixer). Finally, the suggested cleaning procedure was also adequate to prevent cross-contamination between products compounded with the same device.

## Figures and Tables

**Table 1:** Contrast matrix containing factors and levels for the 3^2^ experimental design, with 10 replicates in each level.

Issue	X_1_	X_2_	Responses (*n* = 10)
1	−1	−1	y_1_
2	0	−1	y_2_
3	1	−1	y_3_
4	−1	0	y_4_
5	0	0	y_5_
6	1	0	y_6_
7	−1	1	y_7_
8	0	1	y_8_
9	1	1	y_9_

X_1_ (Mixing time (min)): (−1): 2; (0): 5; (1): 8; X_2_ (Speed (rpm)): (−1): 10; (0): 50; (1): 100.

**Table 2:** Content uniformity for the formulations evaluated in the study.

Formulation/API	Issue	Results (Content of Each Capsule, %)										
		y_1_	y_2_	y_3_	y_4_	y_5_	y_6_	y_7_	y_8_	y_9_	y_10_	AV ^1^
F1/Finasteride	1	102.97	107.14	104.45	102.27	101.73	101.43	103.80	103.82	100.80	100.06	6.3
	2	103.28	101.12	106.16	100.11	101.83	99.49	100.71	97.75	100.01	103.76	5.8
	3	102.59	103.21	103.94	104.93	102.77	106.61	102.69	102.94	108.15	100.80	7.5
	4	104.82	105.63	103.96	100.82	103.75	109.36	100.85	103.74	105.66	102.98	8.6
	5	97.54	102.50	103.48	101.34	104.72	103.24	102.82	101.62	101.71	103.82	5.5
	6	99.90	107.10	105.80	106.78	105.44	102.14	104.24	106.30	102.53	103.89	8.5
	7	101.26	105.61	107.64	100.64	104.26	99.53	99.83	105.49	102.82	102.94	8.0
	8	102.71	101.59	106.15	101.57	97.33	102.29	106.14	104.21	105.99	104.01	8.3
	9	102.25	100.94	100.38	100.03	100.38	99.57	101.73	102.09	101.07	101.37	2.1
F2/Vitamin B12	1	108.86	107.80	107.81	111.07	105.06	112.27	108.36	108.34	109.35	106.38	12.0
	2	107.27	102.41	108.95	106.43	107.41	106.64	105.14	108.88	100.88	108.17	11.2
	3	108.17	105.90	107.21	105.73	103.87	108.05	109.97	107.11	104.00	107.66	9.9
	4	109.46	107.55	109.18	110.48	106.96	102.37	109.54	106.61	103.85	108.45	12.2
	5	110.99	104.26	107.15	106.94	102.25	102.98	109.18	107.85	107.01	108.09	11.8
	6	107.86	112.02	110.96	109.94	109.17	105.07	106.80	110.82	106.51	109.70	12.8
	7	103.33	103.27	100.33	104.42	103.63	107.25	106.93	108.33	105.59	104.85	8.9
	8	99.17	103.46	101.15	100.88	99.93	98.10	98.95	104.91	101.34	101.89	5.0
	9	95.61	93.33	94.44	96.30	96.59	100.97	96.22	98.81	97.32	94.72	7.4
F2/Folic acid	1	95.27	94.87	101.08	97.55	93.66	100.61	101.01	96.00	96.19	95.92	7.8
	2	102.34	103.64	101.76	97.18	97.94	96.64	96.72	97.88	97.11	98.34	6.2
	3	97.05	100.77	102.07	98.66	99.41	99.26	103.62	101.69	97.78	101.31	5.0
	4	104.06	103.37	95.35	94.46	103.17	103.79	95.57	100.74	103.49	101.03	9.3
	5	93.83	103.09	100.32	100.22	100.22	94.95	92.32	98.87	94.78	100.43	9.3
	6	86.77	91.27	86.70	93.13	87.05	91.99	93.55	94.54	94.77	93.32	15.0
	7	82.88	83.28	91.98	88.66	89.08	90.04	85.95	86.47	91.25	86.94	18.3
	8	105.17	98.47	102.37	100.46	93.73	95.53	97.19	98.21	99.42	96.12	8.1
	9	98.73	103.12	98.57	107.43	95.91	95.97	99.55	99.66	96.05	100.57	8.6
F3/Copper chelate	1	98.03	107.10	101.49	106.79	102.77	110.14	106.97	110.37	110.11	108.95	14.8
	2	96.88	103.34	108.32	102.08	102.56	100.95	103.49	98.16	111.25	111.86	14.6
	3	99.52	105.68	106.60	112.42	111.87	104.42	108.31	105.71	105.00	110.34	14.8
	4	106.94	101.07	105.36	106.40	109.24	100.06	107.09	109.39	107.79	102.40	12.0
	5	104.93	104.97	111.32	102.62	105.42	112.68	106.61	102.94	104.95	112.50	14.6
	6	106.02	98.76	99.83	107.57	103.88	107.84	97.15	94.18	110.32	95.74	14.3
	7	101.79	96.85	97.76	93.94	104.67	102.41	96.75	104.04	102.85	96.24	12.9
	8	96.79	98.68	95.83	97.89	100.94	100.66	102.10	103.21	96.59	99.12	6.0
	9	101.20	109.76	105.05	101.56	106.65	103.00	102.51	106.08	104.44	109.35	10.7
F3/Magnesium citrate	1	104.63	109.92	111.41	109.17	107.98	110.27	110.16	108.53	108.78	107.04	11.9
	2	111.81	113.20	111.43	112.20	111.93	107.36	107.86	113.17	109.77	109.37	14.3
	3	110.70	112.02	112.12	106.75	110.78	108.87	111.26	109.61	108.38	107.63	12.8
	4	109.35	107.57	108.94	111.02	112.29	112.48	108.90	108.28	110.80	110.33	12.5
	5	108.18	106.86	108.07	109.47	109.17	110.59	112.38	108.32	112.31	109.16	12.3
	6	112.22	111.92	108.38	111.31	111.02	109.87	110.09	109.23	102.16	107.08	15.0
	7	103.80	109.38	109.14	107.66	108.98	108.87	109.64	110.78	110.29	107.24	11.8
	8	106.71	93.28	94.75	97.87	97.81	99.09	106.68	102.80	105.12	103.08	11.6
	9	108.70	112.69	109.36	111.75	109.72	107.25	108.17	108.25	111.98	107.44	9.2
F3/Zinc chelate	1	95.97	106.20	106.23	104.78	99.55	108.07	106.74	107.73	96.49	105.10	13.2
	2	100.91	105.42	102.71	104.15	106.69	98.96	98.65	105.02	101.47	102.20	7.6
	3	107.77	110.18	111.16	100.44	106.10	104.68	105.85	104.01	102.81	102.08	12.3
	4	106.70	102.99	101.59	100.70	105.41	108.76	104.80	103.43	104.70	106.99	9.1
	5	111.05	112.22	104.10	103.41	104.20	102.13	105.27	96.15	103.61	103.47	13.8
	6	102.23	102.55	107.74	105.41	104.29	101.47	94.34	109.15	106.03	97.05	12.6
	7	107.47	105.78	94.74	108.95	103.07	98.53	101.57	100.98	111.96	100.85	14.3
	8	92.32	94.38	93.16	94.17	91.28	89.95	95.89	97.69	89.95	93.50	11.2
	9	106.30	106.61	111.95	103.34	107.29	105.04	103.19	103.81	105.23	110.23	11.6

^1^ AV: acceptance value.
